# Trends in emergency department visits for mental health disorder diagnoses before and during the COVID-19 pandemic: a retrospective cohort study 2018–2021

**DOI:** 10.1186/s12888-022-03988-y

**Published:** 2022-06-03

**Authors:** Majed Ramadan, Alaa M. Fallatah, Yara F. Batwa, Ziyad Saifaddin, Mohammed S. Mirza, Mona Aldabbagh, Noara Alhusseini

**Affiliations:** 1 King Abdullah International Medical Research Center, Jeddah, Saudi Arabia; 2 King Saud bin Abdulaziz University for Health Sciences, Jeddah, Saudi Arabia; 3 C9F6+JRH, King Abdul Aziz Medical City, Jeddah, 22384 Saudi Arabia; 4 Collage of Medicine, King Abdulaziz University, Jeddah, Saudi Arabia; 5 C9F6+JRH, King Abdul Aziz Medical City, College of Medicine, Jeddah, 22384 Saudi Arabia; 6 Forensic Medicical Center, Ministry of health, Jeddah, Saudi Arabia; 7 Department of Pediatrics, Division of Infectious Diseases, King Abdulaziz Medical City-Jeddah, P.O. Box: 65362, Jeddah, 21556 Saudi Arabia; 8 College of medicine, Alfaisal University, Takhasusi Road, Riyadh, Saudi Arabia

**Keywords:** COVID19, Mental health disorder, Emergency, Anxiety, Depression

## Abstract

**Background:**

As the pandemic started, Saudi Arabia applied extreme measures in responses to the pandemic crises, which have adversely affected individuals’ mental health. Therefore, the aim of this study is to describe trends in mental health disorder ED visit before and after the pandemic in two medical centers in Saudi Arabia.

**Methods:**

A retrospective cohort study using data from electronic health records in the Ministry of National Guard Health Affairs’ (MNG-HA) in Saudi Arabia. Multiple logistic regression model was used to examine the age and sex differences in mental health disorder ED visits before and during the COVID19 pandemic.

**Results:**

A total of 1117 ED psychiatric visits, a sharp increase in the number of psychiatric ED visits during the pandemic with an average increase of 25.66% was observed. During the pandemic, psychiatric ED visits were significantly higher in females (adjusted AOR 1.41, 95% CI 1.08, 1.83) than males. During the COVID19 pandemic, generalized anxiety disorder was the most common psychiatric ED disorder with reported increase of visits by 53.34% (*p-*value 0.005).

**Conclusion:**

ED psychiatric visits have consistently increased before and during the COVID19 pandemic. Interventions for mental health related disorders are urgently needed for preventing mental health problems among females.

## Background

The rapid spread of disease and escalated deaths during global outbreaks of transmissible diseases are often associated with negative psychological impact such as fear and grief [1]. The world experienced historical strict measures following COVID-19 pandemic as social gathering restrictions, shutdown of schools and businesses, curfew, and other measures to prevent the COVID-19 virus spread and to mitigate its impact. These unusual circumstances left millions of individuals isolated, unemployed, and economically disturbed which led to increased risk for mental health problems [2]. Moreover, for many individuals, the COVID19 pandemic can be a traumatic event which is significantly associated with negative psychological consequences at population levels [3]. Globally, mental health disorders including depression are leading cause of disabilities [4]. Before the COVID19 pandemic, increasing burden of mental health disorder was a major public health concern in the Middle East including Saudi Arabia [5]. In 2017 major depressive disorder and anxiety disorder ranked the fourth and fifth leading cause of living with disability among Saudis [5, 6]. In 2018 a cross sectional study conducted in the capital city of Riyadh found that 28% of individuals visiting primary healthcare were diagnosed with a mental health disorder [7]. Despite the huge impact on the health of the population, its well-known that mental health services have been always under-resourced, and least invested in comparison to other health conditions [5–7].

As the COVID19 pandemic started, many countries in the world including Saudi Arabia, applied extreme measures in responses to the public health crises which resulted in a dramatic slow spread of the virus across the country [8]. However, these control measures have adversely affected individuals as many reported psychological illnesses such as depression, anxiety, post-traumatic stress disorder and cognitive deficits [9]. Few studies assessed the impact of COVID − 19 measures on mental health among Saudi population [9–11]. A cross-sectional study estimated the prevalence of anxiety-depression and distress among Saudi population following exposure to the COVID-19 outbreak. Elhessewi’s et al. study concluded that 19.8 and 22.0% of respondents reported moderate to severe anxiety and depression symptoms, respectively [10]. Similarly, Al Mutair et al. conducted a cross-sectional study with more than five thousand Saudi respondents. The study concluded that the COVID19 pandemic negatively impacted individuals’ emotional wellbeing and mental health status [12].

Healthcare professionals along with decisionmakers in Saudi Arabia and around the world, recognize the magnitude of the impact of the pandemic on the mental health population. Globally, the World Health Organization was among the first to respond since the early days of the pandemic. They encouraged all governments to prioritize mental health care and providing more support for population in need [13]. Nationally, this alarming situation have led the Saudi Centers for Disease Control and Prevention (CDC) for the first time in the nation, to conduct a repeated cross-sectional national-level study for mental health screening via computer-assisted phone interviews to identify, track, and monitor trends of the populations at risk of mental illnesses [14]. Additionally, in response to the alarming rise in the mental illness status, the Saudi CDC have initiated several measures to address risk factors for mental health disorders associated with COVID-19 [10]. They also provided stress management guideline to public with focus on vulnerable populations such as children and elderly [10]. However, despite the tremendous advantage of the current mental health surveillance system with high response rate of 81.3%, and previously conducted studies, all of the cases were self-reported and were not confirmed by health professionals. Questions about mental health symptoms might be predictive of but do not necessarily reflect a clinical diagnosis. In the current study, to better understand the potential impact of COVID-19 on mental health, the most common diagnosed mental health disorder, and to determine population at risk. The study describes trends in proportions of several mental health disorder at Emergency department (ED) visits for those who sought urgent mental health services from 2018 to 2021. We further examined trends for each mental health disorder of the study period before and during the pandemic. Finally, we examined sex and age differences in mental health ED visits before and during the COVID19 pandemic in two major cities in Saudi Arabia, Riyadh, and Jeddah.

## Methods

### Study design and setting

The current study is a retrospective cohort (chart review) study using multi center data from the Ministry of National Guard Health Affairs’ (MNG-HA) in Riyadh and Jeddah. King Abdulaziz Medical City (KAMC) in Riyadh and Jeddah with a 1501-bed and 751-bed capacity respectively that provide medical care services in the Central and Western Region of the Kingdom. The medical cities provide all types of care for all National Guard soldiers, their dependents, and individuals reside in Saudi Arabia, starting from primary health care up to tertiary specialized care. KAMC in Riyadh includes 300 ED beds, and Jeddah includes 150 ED beds. Riyadh and Jeddah are the major and largest two cities in Saudi Arabia, KAMC’s Emergency Departments in the two cities include adult and pediatric emergency department that accounts for approximately 25 to 35% of all Saudi Arabia ED visits [15, 16].

### Study population and variables

There were 1117 ED visits for patients diagnosed with mental health disorder from January 2018 to May 31, 2021. Inclusion criteria was individuals aged ≥14 years diagnosed with mental disorder from 2018 to 2021, who were resident in Saudi Arabia and registered in MNG-HA hospitals system as an ED visit. Individuals with duplicate, multiple visits or different location visits, outpatient visit, or inpatient were excluded from the analysis. Since the medical centers use International Classification of Diseases (ICD-10) which have many categories of mental health disorders, we deiced to retrieve the data using the following codes (F409, F410, F411, F419, F431, F319, F329, F29, F322, F39, F20, F42 and F432) for all patient ED visits and diagnosed with adjustment disorder, anxiety disorder, unspecified, generalized anxiety disorder, psychosis, schizophrenia, mood disorders, depression/depressive disorder and major depressive disorder, post-traumatic stress disorder, obsessive-compulsive, panic disorder, phobia, and bipolar. We used date of visit in years, age, sex, nationality, patient type and region as independent variables in our analysis.

### Data sources

Data was extracted from the administrative electronic health records system BestCare at King Abdulaziz Medical City (KAMC). The Ministry of National Guard- Health Affairs (MNG-HA) population include military service personnel and their dependents, members of civilian workforce and students from the MNG-HA related healthcare system. However, EDs in all MNG-HA hospitals are open for the public. Available data include demographics, diagnoses, (using codes from International Classification of Disease ICD-10) and visit characteristics. The ethics committee of King Abdullah International Medical Research Center (KAIMRC) approved this study (JED-21-427,780-99,596). No informed consent was needed due to the retrospective, observational nature of the study.

### Data analysis

Annual mental health disorder ED visits count, and rate were calculated as proportions (number of mental health disorder ED visits over total mental health disorder ED visits) and counts by using data extracted from the health electronic records data for both Riyadh and Jeddah. We used Kruskal–Wallis statistical test to evaluate the overall ED mental health disorder visits differences in proportions across the 2018 through 2021. To understand changes before and during the COVID19 pandemic, we further examined frequency of ED visits before (2018–2019) and during the COVID19 pandemic (2020–2021). For univariate analysis, as normality assumption was violated, we used nonparametric Wilcoxon-Mann-Whitney test to examine the difference of ED visits for each mental health disorder before and during the COVID19 pandemic. We used multiple logistic regression model to examine the age and sex differences in mental health disorder ED visits before and during the COVID19 pandemic. *P*-values were calculated to test whether changes in emergency, mental health visits before and during the COVID19 pandemic differed across demographics. All statistical tests were 2-sided, and findings were considered statistically significant at *P* < .05. All analyses were conducted using SAS statistical software version 9.4 (SAS Institute Inc. Cary, NC).

## Results

**Fig. 1:**
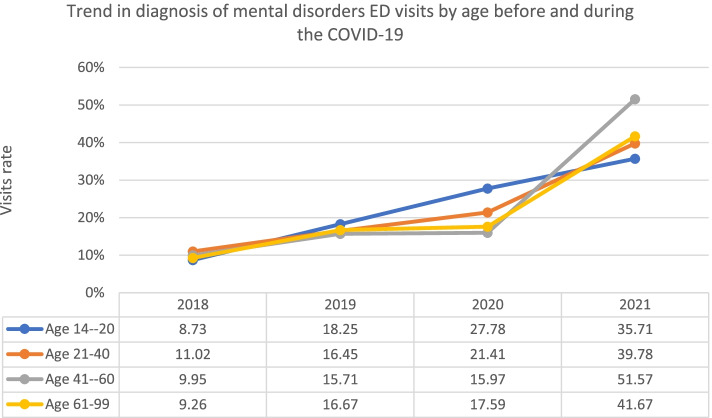
Trend of mental health disorders in ED by age

**Fig. 2:**
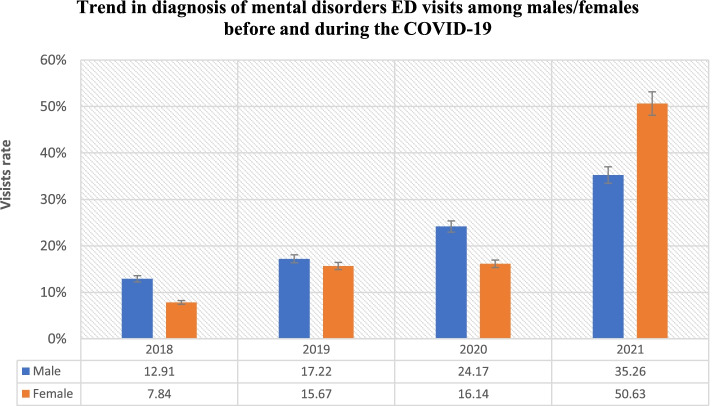
Trend of mental health disorders in ED by gender

Table 1 displays the 4-year trends in mental health disorder ED visits by year. From 2018 to 2021 a total of 1117 ED mental health disorder visits were registered in the health system in Riyadh and Jeddah (51.57% female, 48.43% male). Most of the registered individuals were Saudi national (98.84%) and visited Western region in Jeddah (51.57%). There was 6.8% annual increase in the number of mental health disorder ED visits before the COVID19 pandemic from 2018 to 2019 (11.46, and 18.26%). While the average increase rate during the COVID19 pandemic was 25.66%. Overall mental health disorder visits were highest among age group 21–40 for the entire study period 53.91, 50.49, 53.82, and 46.46% respectively). Group aged (21–40) tended to have higher rate of mental health disorder visits during the COVID19 pandemic (53.82, and 46.46% respectively) than all age groups (Table 1, Fig. 1). There was a significant increase rate in ED psychiatric visits for age group (41–60) during the COVID19 pandemic (36.75%), whereas increase rate for age group (61–99) was 8.4% (Fig. 1). Before the COVID19 pandemic, total mental health disorder visits were higher among males, while in 2021 the percentage of females (60.26%) diagnosed with mental health disorder was higher than males (Table 1, Fig. 2). Nearly half of total mental health disorder visits for the study period occurred during the COVID19 pandemic 2020 through 2021 (47.95%) (Table 1). The total rate of mental disorder ED visits has increased significantly from 11.46% in 2018 to 47.95% in 2021.

**Table 1:** ^1^Number of ED visits for the combined mental health disorders^2^ before and during covid-19 pandemic (*N* = 1117)

		Before		During		***P***-value ^**2**^
		2018	2019	2020	2021	
		n (%)	n (%)	n (%)	n (%)	
Total		128 (11.46)	204 (18.26)	249 (22.29)	536 (47.95)	
**Age**						0.04
14–20	128 (11.46)	11 (8.59)	23 (11.27)	35 (14.06)	45 (8.40)	
21–40	557 (47.57)	69 (53.91)	103 (50.49)	134 (53.82)	249 (46.46)	
41–60	355 (30.32)	38 (29.69)	60 (29.41)	61 (24.50)	197 (36.75)	
61–99	101 (8.63)	10 (7.81)	18 (8.82)	19 (7.63)	45 (8.4)	
**Sex**						<.0001
Male	541 (48.43)	78 (60.94)	104 (50.98)	146 (58.63)	213 (39.74)	
Female	576 (51.57)	50 (39.06)	100 (49.02)	103 (41.37)	323 (60.26)	
**Nationality**						
Saudi	1104 (98.84)	126 (98.44)	201 (98.53)	246 (98.8)	531 (99.07)	0.89
Non-Saudi	13 (1.16)	2 (1.56)	3 (1.47)	3 (1.2)	5 (0.93)	
**Region**						0.2
Central (Riyadh)	541 (48.43)	61 (47.66)	113 (55.39)	138 (55.42)	264 (49.25)	
Western (Jeddah)	576 (51.57)	67 (52.34)	91 (44.61)	111 (44.58)	272 (50.75)	

^1^Data are from King Abdulaziz Medical City (KAMC) in Riyadh and Jeddah, from electronic health records

^2^Mental health disorders include (adjustment disorder, anxiety disorder, unspecified, generalized anxiety disorder, psychosis, schizophrenia, mood disorders, major depressive disorder, depression, depressive disorder, and post-traumatic stress disorder)

^3^Kruskal–Wallis test

^4^No cases were registered

Table 2 reveals changes in ED visits for each mental health disorder. Approximately, one fifth of total ED mental health disorder visits were among patients diagnosed with anxiety disorder during the COVID19 pandemic (18%), then followed by individuals diagnosed with psychosis/schizophrenia (17.91%), depression/depressive disorder, and adjustment disorder 17.5% (Table 2). During the COVID19 pandemic, there was 40.61% increase in total ED mental disorder visits. The highest ED mental disorder visits were among patients diagnosed with panic attack (60%), followed by mood disorder with 62.5% increase, bipolar, phobia with (50%) increase and then depression/depressive disorder/ major depressive disorder (42.64%). The most notable significant difference in number of ED visits was among patients diagnosed with generalized anxiety disorder before and during the COVID19 pandemic (*p*-value 0.005). For other mental health diagnoses, there were no significant difference before and during the COVID19 pandemic (Table 2). In multivariable analysis, there was a statically significant difference between females’ mental disorder ED visits before and during the COVID19 pandemic (AOR 1.41, 95% CI 1.08, 1.83) when compared to males. Females are 1.4 more likely to visit ED during the pandemic compared to males. No other statically significant difference among other covariates was observed (Table 3).

**Table 2:** Numbers and proportions of patients with each mental health diagnostic classification before and during the^1^ pandemic

Mental Disrober Frequency of visits	Before covid-19 2018–2019	During covid-19 2020–2021	Changes	***P*** value ^**2**^
	n (%)	n (%)	(%)	
**Total**			+ 40.61	
**Adjustment disorder**	59 (32.78)	121 (67.22)	+ 34.44	0.48
**Anxiety disorder, unspecified**	57 (31.15)	126 (68.85)	+ 37.77	0.44
**Generalized Anxiety disorder**	28 (23.33)	92 (76.67)	**+ 53.34**	**0.005**
**Post-traumatic stress disorder**	8 (32.00)	17 (68.00)	+ 36	0.75
**Depression/Depressive disorder/ Major depressive disorder**	45 (28.68)	123 (71.32)	+ 42.64	0.64
**Mood disorder**	6 (18.75)	26 (81.25)	+ 62.5	0.45
**Psychosis/ Schizophrenia**	65 (34.88)	126 (65.12)	+ 30.24	0.13
**Bipolar**	12 (25)	36 (75)	+ 50	0.21
**Phobia**	1 (25)	3 (75)	+ 50	0.07
**Panic Attack**	4 (20)	16 (80)	+ 60	0.06
**Obsessive compulsive disorder**	8 (34.78)	15 (65.22)	+ 30.44	0.72

^1^Data are from King Abdulaziz Medical City (KAMC) in Riyadh and Jeddah, from electronic health records

^2^Wilcoxon-Mann-Whitney test for more than 5 frequency per cell

**Table 3:** Multivariate analysis for age and sex differences in mental health disorder ED visits before and during the pandemic^1^

Mental health disrober frequency of visits	Number (rate) of patients before covid-19 2018–2019	Number (rate) of patients during covid-192,020–2021	During vs before covid-19 2020–2021	***P***-value ^**2**^
	n (%)	n (%)	AOR (CI)^**4**^	
**Age group**				
14–20	34 (10.24)	80 (10.19)	1.01 (0.81, 1.68)	0.97
21–40 (ref)^3^	172 (51.81)	383 (48.79)	–	(ref)
41–60	98 (29.52)	258 (32.87)	1.1 (0.95, 1.69)	0.53
61–99	29 (8.43)	64 (8.15)	1.01 (0.62, 1.63)	0.97
**Sex**				
Male (ref)	182 (54.82)	359 (45.73)	–	(ref)
Female	150 (45.18)	426 (54.27)	**1.41 (1.08, 1.83)**	**0.01**
**Nationality**				0.58
Saudi (ref)	5 (1.51)	8 (1.02)	–	(ref)
Non-Saudi	327 (98.49)	777 (98.98)	1.37 (0.43, 4.3)	
**Region**				0.89
Central (Riyadh) (ref)	174 (52.41)	402 (48.79)	–	(ref)
Western (Jeddah)	158 (47.59)	383 (51.21)	0.98 (0.75, 1.27)	

^1^Data are from King Abdulaziz Medical City (KAMC) in Riyadh and Jeddah, from electronic health records

^2^Binary multiple logistic regression was used to examine age/sex differences before and during the pandemic

^3^Reference group = (ref)

^ 4^Adjusted odds ratio AOD and confidence interval CI

## Discussion

To our knowledge, this cohort study is the first to provide hospital encounter data demonstrating a potential association between the COVID-19 pandemic and frequency of mental health disorder ED visits. In the current study, we found that mental health disorder ED visits are increasing constantly across two large medical centers in Saudi Arabia for the years 2018 to 2021 [16]. The study also demonstrated a changing trend of age and sex predominance to ED visits during the COVID19 pandemic, where there was a surge in ED visits during the COVID19 pandemic for those aged 41 to 60. In addition, females became significantly associated with higher odds of mental health disorder ED visits compared to dominance of males before the COVID19 pandemic. Furthermore, the most common diagnosed mental health disorder rate in ED visits during the COVID19 pandemic was generalized anxiety disorder.

The current study found that age group 21–40 years old, are a population with most frequent mental health disorder ED visits before and during the COVID19 pandemic. This finding is consistent with Alosaimi’s study [17] where more than half of the study population were < 40 years old. Furthermore, mental disorders before the COVID19 pandemic were most prevalent among individuals aged < 40 years old [17]. This is expected as approximately 40% of Saudi population are between age 15 to 40 years old [18]. The same patterns were observed among males where a greater number of ED psychiatric visits before the COVID19 pandemic were made by males compared to females. On a global level, before the COVID19 pandemic males also tended to visit ED for mental disorders more frequently than women. In Europe and Switzerland, a large nationally representative data examining ED visits for mental health problems in 2015 found that males represented 75% of ED visits [19]. Similarly, in the U. S a study used the National Hospital Ambulatory Medical Care Survey data from 2014 to 2016 to investigate ED visits. The study found that males were more likely to visit ED for mental disorders than females [20]. However, previously mentioned studies applied different sampling methodologies, and instruments to examine their populations, in addition to the variation in demographic characteristics across these populations. Therefore, they might be heterogeneous to be compared with our population.

It should be noted that the findings were heavily driven by late 2020 and early 2021, in which the largest increase in visits was observed, where the average increase rate of mental health ED visits during this period was three times higher than before the COVID19 pandemic. Nevertheless, these data reflect a continuation of historical trends in mental health disorder ED visits and are in line with recent alarming increases risk of major depressive disorder, and generalized anxiety disorder according to the Saudi national repeated cross-sectional study [14]. This finding is conflicted with Pikkel and Holland’s studies [21, 22] where a significant decrease of ED mental health disorder visits was observed during the pandemic. However, these studies were conducted at the early stages of the COVID19 pandemic where the fear of the COVID19 pandemic was the most and less information was available about the infection and its transmission [22]. Moreover, during the mandatory quarantine, public transportation was shut down at night, and strict rules in the hospital were applied to protect public from the infection [21]. These rules were eased in 2021 in most countries in the world as the vaccines raveled for the public [23]. These facilitations along with partial reopening businesses and services, allowed those who are in urgent need for mental health services to visit emergency department [24]. Thus, studies conducted after the end of first COVID19 wave, or late 2020 and early 2021 suggested the overall ED visits rose during deconfinement to a level never observed before [24–27].

During the COVID19 pandemic, our finding indicates that the largest rise in psychiatric visits were found among age 41 to 60. The main reason for this seems to be that this age group are concerned over the future consequences and economic challenges caused by the COVID19 pandemic, as they are key element in working forces and are, therefore, mostly affected by economic burden and business closures [27, 28]. While male have higher frequency of ED mental health disorder visits for five sequence years before the COVID19 pandemic, female have exceeded the number of ED psychiatric visits during the COVID19 pandemic. This finding indicates that female was at higher risk of seeking mental health disorder emergency assistance during the pandemic than male. One reason for this could be that compared to male, females are more vulnerable to stress and post-traumatic stress disorder as the COVID19 Pandemic [28, 29]. This finding is consistent with previous studies that found the prevalence of anxiety, depression, and stress during COVID-19 pandemic is shown to be higher in female than in male [29–31]. In the early phase of the pandemic, the Center for Diseases Control and Prevention (CDC) using the National Syndromic Surveillance Program (NSSP) data, observed that females had a higher proportion of mental problems ED visits than males [25]. This finding confirms that the prevalence of mental illness among females was greater than in males, this fact should be acknowledged by policymakers and healthcare professionals for taking the appropriate actions. However, more relevant research on women mental health needs should be conducted to fill the existing knowledge gaps on mental disorders, and access for women to health care.

The current study shows that among all mental health disorder visits during the COVID19 pandemic, nearly one fourth of patients were diagnosed with depression and generalized anxiety disorder. This finding is confirmed with previously conducted studies, where depression and anxiety were more prevalent among individuals who experienced the lockdown [29, 32]. During the mandatory quarantine, and in some countries a complete curfew and lockdown as in Saudi Arabia, people tended to watch TV news more than usual [33]. The news published on COVID-19 were filled with false information, rumors, and uncertainties about the virus. All together explains why anxiety rise when a person is constantly exposed to the COVID19 pandemic news [28, 32]. Research shows that people who follow the COVID19 pandemic news the most, experience more anxiety [31]. This phenomenon of increased proportions of reported or diagnosed depression and anxiety is observed all over the world. In both China and the United Kingdom, higher percentages of self-reported depression and anxiety have been observed during the COVID19 pandemic among all age groups [34]. These findings may in part be explained by the high perceived risk of COVID19 because of the limited available information about the COVID19 infection and its associated potential health risk at that time [34]. It has been shown in the scientific literature that risk perception for public crises and events, plays a key role in inducing the psychological and behavioral response as a line of human defense [35]. Additionally, a recent study concluded that the perceived risk has shown to be positively associated with depression and anxiety, which might explain the higher prevalence of depression and anxiety in the early phase of the COVID19 pandemic [35].

Our study includes several limitations. First, the data are not nationally representative, and results may not be generalized to EDs not participating in the Riyadh or Jeddah’s KAMC. Second, multiple important confounders such as mental related visit history of the patients, potential comorbidities, COVID − 19 infection status, and socioeconomic status of the patients were not included in the study which might subject to generate confounding bias.

One strength of this study was that we examined data derived from a diagnosis confirmed by health professionals, unlike self-reported studies which might be subject to measurement errors and self-reported bias. Additionally, our sample size was larger than previous observational studies in the same region conducted in psychiatric Eds.

## Conclusion

Our findings suggest that ED psychiatric visit rates have consistently increased before and during the COVID19 pandemic among young and middle age group. The COVID-19 pandemic is associated with changes in ED presentations requiring psychiatric consultation, particularly among females. During the COVID19 pandemic, female at greater risk of seeking mental health ED services than male. The demand for mental disorder emergency care continues to rise, particularly, for depression and generalized anxiety disorder where the most frequent diagnosed disorders were observed. Given the psychological burden caused by the COVID-19 pandemic and associated restrictions like quarantine, more research is required to inform practice and policy makers in order to improve mental health and psychosocial support during, and after the COVID-19 pandemic. Furthermore, interventions for mental health related disorders are urgently needed for preventing and supporting individuals diagnosed with mental health disorders.

## Data Availability

The data that support the findings of this study are available on request from the corresponding author, M.R. The data are not publicly available due to sensitive identifier that have been used in this study, which were used under license for the current study.

## Abbreviations

ED: Emergency Department
COVID19: coronavirus disease of 2019
KAMC: King Abdulaziz Medical City
MNG-HA: The Ministry of National Guard- Health Affairs

## References

[1] Amsalem D, Dixon LB, Neria Y (2021). The coronavirus disease 2019 (COVID-19) outbreak and mental health: current risks and recommended actions. JAMA Psychiatry.

[2] Berkowitz SA, Basu S (2021). Unemployment insurance, health-related social needs, health care access, and mental health during the COVID-19 pandemic. JAMA Intern Med.

[3] Fiorillo A, Sampogna G, Giallonardo V, Del Vecchio V, Luciano M, Albert U, et al. Effects of the lockdown on the mental health of the general population during the COVID-19 pandemic in Italy: results from the COMET collaborative network. Eur Psychiatry. 2020;63(1):e87. 10.1192/j.eurpsy.2020.89. 10.1192/j.eurpsy.2020.89 PMC7556907 32981568

[4] Friedrich MJ (2017). Depression is the leading cause of disability around the world. JAMA.

[5] Tyrovolas S, El Bcheraoui C, Alghnam SA, Alhabib KF, Almadi MAH, Al-Raddadi RM, Mokdad AH (2020). The burden of disease in Saudi Arabia 1990–2017: results from the global burden of disease study 2017. Lancet Planet Health.

[6] World Health Organization (2017). Mental health atlas 2017-member state profile.

[7] Alghadeer SM, Alhossan AM, Al-Arifi MN, Alrabiah ZS, Ali SW, Babelghaith SD, Altamimi MA (2018). Prevalence of mental disorders among patients attending primary health care centers in the capital of Saudi Arabia. Neurosci J.

[8] Algaissi AA, Alharbi NK, Hassanain M, Hashem AM (2020). Preparedness and response to COVID-19 in Saudi Arabia: building on MERS experience. J Infect Public Health.

[9] Elhessewi GMS, Almoayad F, Mahboub S, Alhashem AM, Fiala L (2021). Psychological distress and its risk factors during COVID-19 pandemic in Saudi Arabia: a cross-sectional study. Middle East Curr Psychiatry.

[10] Saudi center for disease prevention and control (CDC) (2020). The new corona virus (COVID-19) preventive guide for mental and social health.

[11] Joseph R, Lucca JM, Alshayban D, Alshehry YA (2021). The immediate psychological response of the general population in Saudi Arabia during COVID-19 pandemic: a cross-sectional study. J Infect Public Health.

[12] Al Mutair A, Alhajji M, Shamsan A (2021). Emotional wellbeing in Saudi Arabia during the COVID-19 pandemic: a National Survey. Risk Manag Healthc Policy.

[13] Santomauro DF, Herrera AMM, Shadid J, Zheng P, Ashbaugh C, Pigott DM, Ferrari AJ (2021). Global prevalence and burden of depressive and anxiety disorders in 204 countries and territories in 2020 due to the COVID-19 pandemic. Lancet.

[14] BinDhim NF, Althumiri NA, Basyouni MH, Alageel AA, Alghnam S, Al-Qunaibet AM, Ad-Dab’bagh, Y. (2021). Saudi Arabia mental health surveillance system (MHSS): mental health trends amid COVID-19 and comparison with pre-COVID-19 trends. Eur J Psychotraumatol.

[15] Alenazi T, Al Arbash H, El-Saed A, Alshamrani M, Baffoe-Bonnie H, Arabi M, Balkhy H (2017). Identified transmission dynamics of Middle East respiratory syndrome coronavirus infection during an outbreak: implications of an overcrowded emergency department. Clin Infect Dis.

[16] Alyabsi M, Ramadan M, Algarni M, Alshammari K, Jazieh AR (2021). The effect of marital status on stage at diagnosis and survival in Saudis diagnosed with colorectal cancer: cancer registry analysis. Sci Rep.

[17] Alosaimi FD, Alzain N, Asiri S, Fallata E, Abalhassan M, Qrmli A, Alhabbad A (2017). Patterns of psychiatric diagnoses in inpatient and outpatient psychiatric settings in Saudi Arabia. Arch Clin Psychiatry (São Paulo).

[18] Pikkel Igal Y, Meretyk I, Darawshe A, Hayek S, Givon L, Levy A, et al. Trends in psychiatric emergency department visits in northern Israel during the COVID-19 outbreak. Front Psychiatry. 2021;1106. 10.3389/fpsyt.2021.603318 PMC8329092 34354606

[19] Slankamenac K, Heidelberger R, Keller DI (2020). Prediction of recurrent emergency department visits in patients with mental disorders. Front Psychiatry.

[20] Hill T, Jiang Y, Friese CR, Darbes LA, Blazes CK, Zhang X (2020). Analysis of emergency department visits for all reasons by adults with depression in the United States. BMC Emerg Med.

[21] Holland KM, Jones C, Vivolo-Kantor AM, Idaikkadar N, Zwald M, Hoots B, Houry D (2021). Trends in US emergency department visits for mental health, overdose, and violence outcomes before and during the COVID-19 pandemic. JAMA Psychiatry.

[22] Han E, Tan MMJ, Turk E, Sridhar D, Leung GM, Shibuya K (2020). Lessons learnt from easing COVID-19 restrictions: an analysis of countries and regions in Asia Pacific and Europe. Lancet..

[23] Flament J, Scius N, Zdanowicz N, Regnier M, De Cannière L, Thonon H (2021). Influence of post-COVID-19 deconfinement on psychiatric visits to the emergency department. Am J Emerg Med.

[24] Chadi N, Spinoso-Di Piano C, Osmanlliu E, Gravel J, Drouin O (2021). Mental Health–related emergency department visits in adolescents before and during the COVID-19 pandemic: a multicentric retrospective study. J Adolesc Health.

[25] Leeb RT, Bitsko RH, Radhakrishnan L, Martinez P, Njai R, Holland KM (2020). Mental health–related emergency department visits among children aged< 18 years during the COVID-19 pandemic—United States, January 1–October 17, 2020. Morb Mortal Wkly Rep.

[26] Huang Y, Zhao N (2020). Generalized anxiety disorder, depressive symptoms and sleep quality during COVID-19 outbreak in China: a web-based cross-sectional survey. Psychiatry Res.

[27] Salari N, Hosseinian-Far A, Jalali R, Vaisi-Raygani A, Rasoulpoor S, Mohammadi M, Khaledi-Paveh B (2020). Prevalence of stress, anxiety, depression among the general population during the COVID-19 pandemic: a systematic review and meta-analysis. Glob Health.

[28] Lim GY, Tam WW, Lu Y, Ho CS, Zhang MW, Ho RC (2018). Prevalence of depression in the community from 30 countries between 1994 and 2014. Sci Rep.

[29] Moghanibashi-Mansourieh A (2020). Assessing the anxiety level of Iranian general population during COVID-19 outbreak. Asian J Psychiatr.

[30] Ueda M, Stickley A, Sueki H, Matsubayashi T. Mental health status of the general population during the COVID-19 pandemic: a cross-sectional national survey in Japan. MedRxiv. 2020.04.28.20082453. 10.1101/2020.04.28.20082453.

[31] Wu T, Jia X, Shi H, Niu J, Yin X, Xie J, Wang X (2021). Prevalence of mental health problems during the COVID-19 pandemic: a systematic review and meta-analysis. J Affect Disord.

[32] World Health Organization. Mental health and psychosocial considerations during the COVID-19 outbreak, 18 March 2020. World Health Organization; 2020. https://apps.who.int/iris/handle/10665/331490. License: CC BY-NC-SA 3.0 IGO.

[33] McDowell MJ, Fry CE, Nisavic M, Grossman M, Masaki C, Sorg E, Beach SR (2021). Evaluating the association between COVID-19 and psychiatric presentations, suicidal ideation in an emergency department. Plos One.

[34] Robinson E, Sutin AR, Daly M, Jones A (2022). A systematic review and meta-analysis of longitudinal cohort studies comparing mental health before versus during the COVID-19 pandemic in 2020. J Affect Disord.

[35] Li X, Lyu H. Epidemic risk perception, perceived stress, and mental health during COVID-19 pandemic: a moderated mediating model. Front Psychol. 2021;4100. 10.3389/fpsyg.2020.563741 PMC7902491 33643107

